# Distinct Molecular and Prognostic Profiles of Left‐ and Right‐Sided Colorectal Cancer Revealed by NGS Analysis: The Role of *SMAD4* and *SETD2* Mutations

**DOI:** 10.1002/cam4.71534

**Published:** 2026-01-21

**Authors:** Wenlei Zhao, Yuxuan Qiu, Na Bai, Chunhong He, Jiani C. Yin, Qianru Xu, Kaiyu Jian, Baolei Jia, Lin Jiang, Feng Liang

**Affiliations:** ^1^ Department of General Surgery First Medical Center, Chinese People's Liberation Army (PLA) General Hospital Beijing China; ^2^ Geneseeq Research Institute Nanjing Geneseeq Technology Inc. Nanjing China

**Keywords:** colorectal neoplasms, genetic variation, next‐generation sequencing, prognosis, tumor location

## Abstract

**Background:**

Colorectal cancer (CRC) isthe third most common cancer and the second leading cause of cancer‐relateddeath worldwide. Its genetic heterogeneity complicates treatment. This studyaimed to compare clinicopathological, molecular, and prognostic factors betweenleft‐sided (LCC) and right‐sided (RCC) CRC.

**Methods:**

We retrospectively analyzed 48 CRCpatients who received next‐generation sequencing (NGS) of tumor tissue andmatched leukocytes using a 425‐gene panel. Clinicopathological, molecular, andprognostic factors were compared between LCC and RCC.

**Results:**

RCC exhibited a higher frequencyof BRAF mutations (33.3% vs. 7.1%, *p* = 0.042) and more frequent alterations in PI3K (*p* = 0.033), homology‐dependent recombination (HDR, *p* = 0.018), and mismatch repair (MMR, *p* = 0.002) pathways than LCC. Multivariate analysis identified SMAD4 mutation as an independentpredictor of worse overall survival (OS) (HR = 3.88, 95% CI 1.05–14.29, *p* = 0.042), which was confirmed in an external cohort of 1796 CRCpatients. In subgroup analyses, SETD2 mutationswere associated with poor prognosis in LCC (HR = 2.20, 95% CI 0.80–6.06, *p* = 0.026), while ARID1A (*p* = 0.040) and PRDM1 (*p* = 0.021) mutations correlated with shorter progression‐free survival in RCC. Patients harboring both SMAD4 and SETD2 mutationshad the shortest OS (median: 17.7 months, *p* < 0.0001).

**Conclusions:**

These findings reveal criticalmolecular and prognostic differences between LCC and RCC and highlight SMAD4 and SETD2 asimportant prognostic biomarkers, suggesting the potential value of location‐and mutation‐guided precision therapies in CRC.

**Abbreviations**
5‐FU: 5‐fluorouracil
BER: base excision repair
BWA: Burrows‐Wheeler Aligner
CAP: College of American Pathologists
CI: confidence interval
CIMP‐H: high CpG island methylator phenotype
CIN: chromosomal instability
CLIA: Clinical Laboratory Improvement Amendments
CMS‐1: consensus molecular subtype‐1
CNV: copy number variation
CRC: colorectal cancer
DDR: DNA damage response
ECOG: Eastern Cooperative Oncology Group
EGFR: epidermal growth factor receptor
FA: Fanconi anemia
FFPE: formalin‐fixed paraffin‐embedded
GATK: Genome Analysis Toolkit
HDR: homology‐dependent recombination
hg19: Human genome version 19
HR: hazard ratio
IGV: Integrative Genomics Viewer
indel: insertion/deletion
ISO: International Organization for Standardization
LCC: left‐sided colorectal cancer
Mb: megabase
MMR: mismatch repair
mOS: median OS
MS: microsatellite
MSI‐H: high microsatellite instability
MSS: microsatellite stability
NGS: next‐generation sequencing
OS: overall survival
PFS: progression‐free survival
RCC: right‐sided colorectal cancer
SNV: single nucleotide variant
TMB: tumor mutation burden
TMB‐H: high TMB
TMB‐L: low TMB
VAF: variant allele frequency

## 1 Introduction

Colorectal cancer (CRC) ranks as the third most common cancer and the second leading cause of cancer‐related mortality globally [1], underlining the critical challenges in managing CRC. The complexity of CRC is largely driven by its significant heterogeneity, arising from diverse cancerous lesions within the colon or rectum, coupled with its intricate genetic landscape. CRC is anatomically divided into left‐sided (LCC) and right‐sided (RCC) cancers, which differ in embryological origin, histopathology, molecular profiles, and immune context [2]. Recent advances in the treatment of metastatic CRC have integrated chemotherapy with targeted therapies, and the latest developments include immunotherapy via immune checkpoint inhibitors [3, 4, 5]. Despite these therapeutic innovations, the five‐year survival rate for metastatic CRC remains alarmingly below 15% [5].

Heterogeneity between LCC and RCC significantly influences prognosis and response to therapy [6, 7]. Generally, RCC is associated with a poorer prognosis compared to LCC [8, 9, 10, 11]. RCC often harbors *BRAF* mutations, exhibits larger tumor sizes at diagnosis, and shows high microsatellite instability (MSI‐H) and high CpG island methylator phenotype (CIMP‐H) [12, 13, 14]. These tumors are typically categorized as consensus molecular subtype‐1 (CMS‐1) and have shown improved response to immunotherapy, likely due to higher tumor mutational burden (TMB) [15]. Conversely, LCC is more common in males and is often associated with chromosomal instability (CIN), with frequent mutations in *KRAS*, *APC*, *PIK3CA*, and *TP53*. These genetic features make LCC more suitable for treatment with adjuvant chemotherapy, such as 5‐fluorouracil (5‐FU), and therapies targeting the epidermal growth factor receptor (EGFR) [16]. These molecular and clinical distinctions underscore the need for location‐based, personalized treatment strategies.

Against this background, personalized medicine is needed for CRC management. Next‐generation sequencing (NGS) has emerged as an essential tool for comprehensive molecular characterization, facilitating more precise and individualized therapeutic strategies [17, 18]. In this study, we used NGS to analyze 48 primary CRC samples, focusing on LCC and RCC distinctions. We aimed to clarify CRC's molecular diversity and uncover the prognostic significance of key genetic alterations to guide optimized, personalized therapy.

## 2 Material and Methods

### 2.1 Patients and Sample Collection

This retrospective study included CRC patients at the First Medical Center of the Chinese People's Liberation Army (PLA) General Hospital from January 2022 to June 2024. A total of 48 patients met the inclusion criteria: (1) no neoadjuvant chemotherapy or other preoperative treatments; (2) confirmed postoperative pathological CRC; and (3) sufficient tumor tissue and paired white blood cell samples for NGS. Comprehensive demographic and clinical information, including gender, age, family history, distal metastasis, Eastern Cooperative Oncology Group (ECOG) performance status, and diagnostic stage, were collected. Tumors were categorized as RCC for those located in the cecum, ascending colon, hepatic flexure, or transverse colon, and LCC for those in the splenic flexure, descending colon, sigmoid colon, or rectum. Microsatellite stability (MSS) and instability (MSI) were assessed using an NGS panel. Survival data were recorded with telephone interviews. Primary endpoints were overall survival (OS, time from diagnosis to death or last follow‐up) and progression‐free survival (PFS, time from diagnosis to progression or death). The study was approved by the Ethics Committee of the PLA General Hospital, and informed consent was obtained.

### 2.2 DNA Extraction and Library Construction

All collected samples were subjected to NGS using a pan‐cancer panel of 425 genes (GeneseeqPrime) in a College of American Pathologists (CAP)‐accredited, Clinical Laboratory Improvement Amendments (CLIA)‐certified, and International Organization for Standardization (ISO) 15,189‐accredited laboratory (Nanjing Geneseeq Technology Inc.) following established protocols [19]. The full list of genes is provided in Table S1. Briefly, this comprehensive panel covers critical genomic regions relevant to CRC biology, including key driver genes such as *APC*, *TP53*, *KRAS*, *BRAF*, *SMAD4*, and major signaling pathways such as WNT, RAS/MAPK, PI3K/AKT, and TGF‐*β*, as well as genes involved in DNA damage response (DDR). Genomic DNA from formalin‐fixed, paraffin‐embedded (FFPE) samples was extracted using the QIAamp DNA FFPE Tissue Kit (Qiagen, Hilden, Germany), and DNA from white blood cells was extracted using the DNeasy Blood and Tissue Kit (Qiagen, Hilden, Germany). DNA quality and quantification were assessed by a Nanodrop 2000 (Thermo Fisher Scientific, Waltham, USA) and a Qubit 3.0 fluorometer (Life Technologies, Carlsbad, USA), respectively. Library preparation was conducted with the KAPA HyperPrep Kit (KAPA Biosystems, Woburn, USA) according to the optimized protocol, achieving mean sequencing depths of 500× for tumor tissue and > 100× for matched white blood cells (as germline controls), as previously described [19]. Libraries were processed, followed by amplification, and quantified by qPCR.

### 2.3 Data Processing and Mutation Calling

Sequencing was performed on the Illumina HiSeq4000 platform. Initial demultiplexing was accomplished with bcl2fastq version 2.19. Trimmomatic [20] was used for quality control of FASTQ files to eliminate low‐quality reads or *N* bases. High‐quality reads were then aligned to the Human genome version 19 (hg19) reference using Burrows‐Wheeler Aligner (BWA, version 0.7.12; https://github.com/lh3/bwa/tree/master/bwakit). Local realignment and base quality score recalibration were performed using the Genome Analysis Toolkit (GATK 3.4.0; https://software.broadinstitute.org/gatk/). Somatic mutations, including single nucleotide variants (SNVs) and insertions/deletions (indels), were identified using VarScan2 (https://varscan.sourceforge.net/) [21] and annotated using ANNOVAR (https://annovar.openbioinformatics.org/en/latest/) [22]. Variants with a variant allele frequency (VAF) ≥ 0.01 were considered, while common variants with frequency > 1% from the 1000 Genomes Project and dbSNP were excluded. An internal recurrent artifact list was used to further screen mutations, and paired white blood cells were sequenced to remove sequencing artifacts, germline variants, and clonal hematopoiesis. Genomic fusions were identified by DELLY [23] with default parameters, and manually reviewed via the Integrative Genomics Viewer (IGV, version 2.13.2) [24]. Copy number variations (CNVs) were identified using CNVkit with default parameters [25]. Depth ratios of above 2.0 and below 0.6 were considered as CNV gain and CNV loss, respectively [26].

### 2.4 Tumor Mutational Burden, Microsatellite Status, and Pathway Analysis

TMB was calculated as the number of somatic, coding, base substitution, and indel mutations (excluding known driver mutations) per megabase (Mb) of genome, as previously described [27]. Tumors with ≥ 10 mutations/Mb were classified as high TMB (TMB‐H). Microsatellite (MS) status was determined on the overall stability of MS loci covered by the GeneseeqPrime panel using a proprietary in‐house developed MSI analysis pipeline, as previously reported [27]. The classification of MSI‐H or MSS was achieved by setting thresholds according to varying sequencing depths, allowing for robust differentiation between the two statuses. In addition, six colorectal cancer‐related pathways, encompassing a total of 47 genes, were analyzed based on established literature [6, 16, 28].

### 2.5 External Validation of the Prognostic Value of SMAD4 and SETD2 Alterations

The prognostic significance of *SMAD4* and *SETD2* alterations in CRC was validated using data on 1796 CRC patients obtained from the cBioportal database (https://www.cbioportal.org). Patients were divided into subgroups based on the presence or absence of *SMAD4 and SETD2* alterations to investigate their correlations with survival outcomes. Both univariate and multivariate Cox regression analyses were employed to assess the prognostic value of these genetic alterations.

### 2.6 Statistical Analysis

Categorical variables were compared using Fisher's exact test. Continuous variables were described as medians with ranges and compared using the Wilcoxon rank‐sum test. Survival was analyzed by Kaplan–Meier and log‐rank test, and Cox regression was used for univariate and multivariate analyses, with hazard ratios (HRs) and 95% confidence intervals (CIs) reported. Variables with *p*‐values < 0.15 in univariate analysis were subsequently included in the multivariate models. A two‐sided *p*‐value < 0.05 was considered statistically significant (*p < 0.05, **p < 0.01, ***p < 0.001, ****p < 0.0001). All analyses were performed using R (version 3.6.1).

## 3 Results

### 3.1 Clinicopathologic and Molecular Characteristics of the Study Cohort

Our study included 48 patients diagnosed with colorectal carcinoma who underwent surgical resection. To ensure cohort robustness and minimize potential confounding factors, we conducted a meticulous case‐by‐case review of molecular profiles, confirming that all included patients met the strict criteria for sporadic CRC. Baseline clinicopathologic and molecular features are summarized in Table 1. The median age was 64 years (range, 38–90), and 64.6% were male. Most patients had ECOG 0–1 (87.5%) and advanced stage (stage III or IV, 66.7%). Moderate and poor tumor differentiation was observed in 37.5% of cases. MSI‐H and TMB‐H were noted in 10.4% and 14.6% of the patients, respectively. In terms of molecular alterations, 41.7% of patients were RAS (*KRAS* and *NRAS*) wild‐type, and 83.3% lacked *BRAF* mutations. Tumor location was left‐sided in 58.3%, right‐sided in 37.5%, and mixed in 4.2% of cases (Table 1).

**TABLE 1:** Comparison of clinicopathologic and molecular characteristics between LCC and RCC.

Characteristic	All (*n* = 48)	LCC (*n* = 28)	RCC (*n* = 18)	*p*
Age, *n* (%)				
≥ 60	30 (62.5)	15 (53.6)	13 (72.2)	0.234
< 60	18 (37.5)	13 (46.4)	5 (27.8)	
Sex, *n* (%)				
Male	31 (64.6)	22 (78.6)	8 (44.4)	0.027*
Female	17 (35.4)	6 (21.4)	10 (55.6)	
Differentiation, *n* (%)				
Poorly differentiated	7 (14.6)	6 (21.4)	1 (5.6)	0.090
Poorly‐moderately differentiated	11 (22.9)	3 (10.7)	7 (38.9)	
Moderately differentiated	21 (43.8)	13 (46.4)	8 (44.4)	
Unknown	9 (18.7)	6 (21.4)	2 (11.1)	
Pathological stage, *n* (%)				
I	4 (8.3)	2 (7.1)	2 (11.1)	0.619
II	12 (25.0)	7 (25.0)	4 (22.2)	
III	20 (41.7)	10 (35.7)	9 (50.0)	
IV	12 (25.0)	9 (32.1)	3 (16.7)	
ECOG score, *n* (%)				
0–1	42 (87.5)	26 (92.9)	14 (77.8)	0.191
2/other	6 (12.5)	2 (7.1)	4 (22.2)	
Treatment, *n* (%)				
Chemo alone	24 (50.0)	15 (53.6)	9 (50.0)	0.471
Chemo + bevacizumab	9 (18.7)	6 (21.4)	2 (11.1)	
Chemo + cetuximab	1 (2.1)	1 (3.6)	0 (0)	
No treatment	14 (29.2)	6 (21.4)	7 (38.9)	
MS status, *n* (%)				
MSI‐H	5 (10.4)	0	5 (27.8)	0.006**
MSS	43 (89.6)	28 (100)	13 (72.2)	
TMB, *n* (%)				
TMB‐H	7 (14.6)	2 (7.1)	5 (27.8)	0.093
TMB‐L	41 (85.4)	26 (92.9)	13 (72.2)	
RAS, *n* (%)				
Mutant type	28 (58.3)	14 (50.0)	12 (66.7)	0.364
Wild type	20 (41.7)	14 (50.0)	6 (33.3)	
*BRAF*, *n* (%)				
Mutant type	8 (16.7)	2 (7.1)	6 (33.3)	0.042*
Wild type	40 (83.3)	26 (92.9)	12 (66.7)	
*BRAF* V600E, *n* (%)				
Mutant type	4 (8.3)	2 (7.1)	2 (11.1)	0.639
Wild type	44 (91.7)	26 (92.9)	16 (88.9)	

*Note:* Data was analyzed using a Fisher's exact test.

Abbreviations: Chemo, chemotherapy; ECOG, Eastern Cooperative Oncology Group; LCC, left‐sided colorectal cancer; MS, microsatellite status; MSI‐H, high microsatellite instability; MSS, microsatellite stable; RCC, right‐sided colorectal cancer; TMB, tumor mutation burden; TMB‐H, high tumor mutation burden; TMB‐L, low tumor mutation burden.

** < 0.01.

* < 0.05.

### 3.2 Molecular Landscape and Interaction of the CRC Study Cohort

**FIGURE 1:**
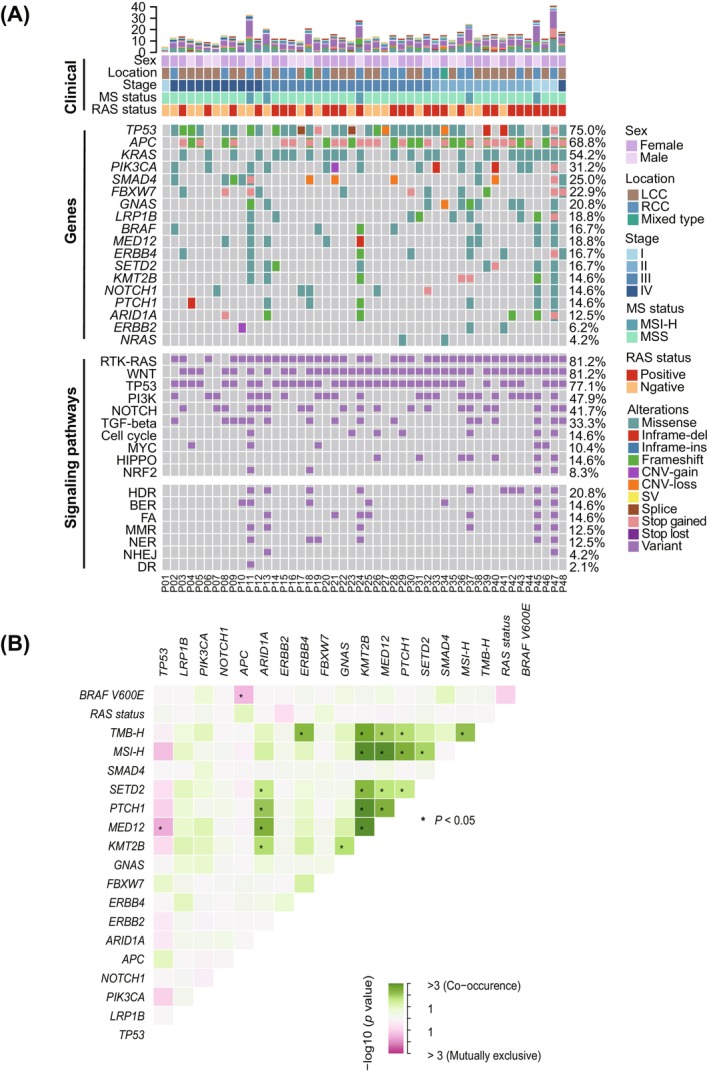
Comprehensive molecular landscape of colorectal cancer in the study cohort. (A) The mutational landscape of colorectal cancer (CRC) patients. Top bars indicate clinicopathologic subtypes of patients, while the right‐side color legend distinguishes between different mutation types. Each column represents an individual patient in the cohort. (B) Interaction analysis of high‐frequency mutations and bioinformatic features in CRC patients (*n* = 48). The *x*‐axis and *y*‐axis represent different genes and bioinformatic features. Interactions are visualized using a color gradient, with red indicating exclusivity and green indicating occurrence. Statistical significance was determined by Fisher's exact test, and *p*‐values less than 0.05 (labeled in “*”) after the FDR correction were considered significant. BER, base excision repair; CNV, copy number variation; DR, direct repair; FA, Fanconi anemia; HDR, homology‐dependent recombination; LCC, left‐sided colorectal cancer; MMR, mismatch repair; MS, microsatellite; MSI‐H, high microsatellite instability; MSS, microsatellite stability; NER, nucleotide excision repair; NHEJ, non‐homologous end joining; RCC, right‐sided colorectal cancer; SV, structural variation.

We further characterized the molecular landscape to clarify underlying genetic alterations. We observed a high prevalence of missense mutations (71.9%), followed by frameshift mutations (11.8%, Figure 1A). The most frequently mutated genes were *TP53* (75%), *APC* (68.8%), *KRAS* (54.2%), and *PIK3CA* (31.2%). These mutations were mainly enriched in key oncogenic pathways, including RTK‐RAS (81.2%), WNT (81.2%), TP53 (77.1%), and PI3K (47.9%) signaling pathways. Moreover, mutations were also enriched in the DDR pathway, specifically in homology‐dependent recombination (HDR, 20.8%), base excision repair (BER, 14.6%), and Fanconi anemia (FA, 14.6%) pathways (Figure 1A).

To further explore the genetic interactions in CRC, we performed the analysis of co‐occurrence and mutual exclusivity among genes and pathways (Figure 1B). Significant mutual exclusivity was identified between *MED12* and *TP53* (*p* < 0.05 after FDR adjustment). Although MSI‐H was observed to be mutually exclusive with *TP53*, this association did not reach statistical significance (*p* > 0.05). Importantly, *BRAF* V600E was mutually exclusive with both RAS status and *APC* alterations (*p* < 0.05). Furthermore, TMB‐H co‐occurred with alterations in *ERBB4*, *MED12*, *KMT2B*, *PTCH1*, and MSI‐H, whereas MSI‐H co‐occurred significantly with alterations in *MED12*, *KMT2B*, *PTCH1*, and *SETD2* (*p* < 0.05, Figure 1B).

### 3.3 Comparison of Clinicopathologic and Molecular Characteristics Between LCC and RCC

In the subgroup analysis excluding the two mixed‐type cases (*n* = 46), LCC patients were more often male (78.6% vs. 44.4%, *p* = 0.027). Additionally, MSI‐H status was significantly more prevalent in RCC (27.8%) than in LCC (0%, *p* = 0.006). Molecular profiling showed a higher frequency of *BRAF* mutations (33.3%) in RCC compared to LCC (7.1%, *p* = 0.042). However, no significant differences were found in overall RAS mutation status between LCC and RCC (*p* = 0.364). No additional clinicopathologic or molecular features showed a significant association with tumor location (*p* > 0.05, Table 1). Importantly, treatment regimens (including chemotherapy alone or in combination with targeted therapies) were balanced between the LCC and RCC groups (*p* = 0.471, Table 1), indicating no regimen‐based bias in patient stratification.

### 3.4 Comparison of Genetic Landscape Between LCC and RCC

**FIGURE 2:**
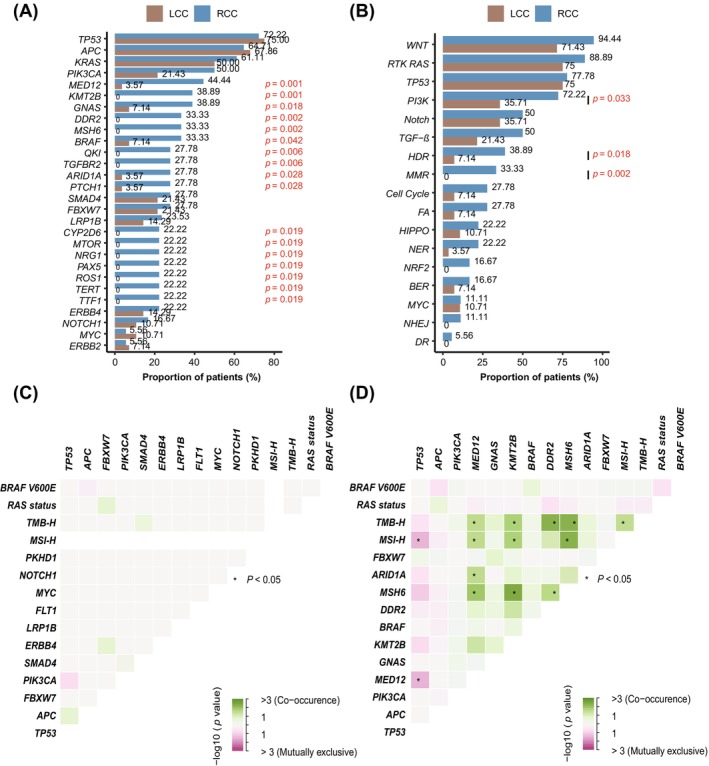
Distinct molecular landscapes of LCC and RCC. (A, B) Comparison of frequencies of high‐frequency gene mutations (A) and gene pathway alterations (B) in LCC versus RCC tumors. The *x*‐axis represents the proportion of patients, and the *y*‐axis lists different gene pathways. Bar heights and colors correspond to mutation frequencies. Statistical significance was determined by Fisher's exact test, and *P*‐values less than 0.05 were considered significant. (C, D) Interaction analysis of high‐frequency mutations and bioinformatic features in LCC patients (*n* = 28) (C) and in RCC patients (*n* = 18) (D). The *x*‐axis and *y*‐axis represent different genes and bioinformatic features. Interactions are visualized using a color gradient, with red indicating exclusivity and green indicating occurrence. Statistical significance was determined by Fisher's exact test, and *p*‐values less than 0.05 (labeled in “*”) after the FDR correction were considered significant. BER, base excision repair; DR, direct repair; FA, Fanconi anemia; HDR, homology‐dependent recombination; LCC, left‐sided colorectal cancer; MMR, mismatch repair; NER, nucleotide excision repair; NHEJ, non‐homologous end joining; RCC, right‐sided colorectal cancer.

Expanding upon the established heterogeneity of CRC, we examined the specific genetic alterations or pathways characterizing LCC and RCC. Despite sharing frequently mutated genes such as *TP53*, *APC*, and *KRAS* (Figure S1A), differences were identified between the two subgroups. Only 29.3% of gene alterations were identical between LCC and RCC, while a considerable number of mutations were location‐specific, with a greater predominance in RCC (66.7%, Figure S1B). Several genes exhibited either significantly higher mutation rates or were exclusively mutated in RCC compared to LCC, including *MED12*, *GNAS*, *ARID1A*, *MSH6*, and *BRAF* (*p* < 0.05, Figure 2A). Pathway analysis further demonstrated significantly higher rates of alterations of PI3K (72.2% vs. 35.7%, *p* = 0.033), HDR (38.9% vs. 7.1%, *p* = 0.018), and mismatch repair (MMR, 33.3% vs. 0%, *p* = 0.002) pathways (Figure 2B). Detailed distributions of PI3K, HDR, and MMR pathway mutations in LCC and RCC are presented in Figure S1C.

No significant genetic interactions were observed in LCC, whereas RCC exhibited a complex network of genetic interactions (Figure 2C). The *MSH6* mutations co‐occurred with both TMB‐H and MSI‐H in RCC (*p* < 0.05 after FDR adjustment). Moreover, *MED12* mutations were significantly associated with TMB‐H, MSI‐H, *ARID1A*, and *MSH6* alterations (*p* < 0.05 after FDR adjustment). Furthermore, mutual exclusivity was identified between *TP53* and MSI‐H, as well as *TP53* and *MED12* (*p* < 0.05 after FDR adjustment, Figure 2D).

### 3.5 Relationship Between Clinical and Genetic Factors and Prognosis

Univariate analysis of the overall cohort revealed that advanced TNM stage (III/IV) was associated with a significantly increased risk of disease progression compared to early‐stage disease (I/II) (HR = 4.36, 95% CI 0.99–19.20, *p* = 0.034). Lung metastasis was significantly associated with disease progression (HR = 4.57, 95% CI 1.02–20.56, *p* = 0.030), and poorer OS (HR = 7.31, 95% CI 1.54–34.76, *p* = 0.003, Table 2). Analysis of treatment regimens revealed no difference in PFS (*p* = 0.422) or OS (*p* = 0.993), ruling out therapeutic variance as a prognostic driver (Table 2).

**TABLE 2:** The prognostic relevance of clinical characteristics in the study cohort.

Characteristics	PFS		OS	
	HR (95% CI)	*p*	HR (95% CI)	*p*
TNM stage				
III + IV versus I + II	4.36 (0.99–19.20)	0.034*	5.28 (0.67–41.74)	0.078
Treatment				
Combination versus chemo alone	1.57 (0.51–4.82)	0.422	0.99 (0.19–5.12)	0.993
Liver metastasis				
M1 versus M0	1.92 (0.44–8.47)	0.381	4.159 (0.88–19.68)	0.051*
Lung metastasis				
M1 versus M0	4.57 (1.02–20.56)	0.030*	7.31 (1.54–34.76)	0.003**
*SMAD4*				
Mutant versus wild	2.20 (0.80–6.06)	0.118	3.68 (1.06–12.73)	0.027*
*AMER1*				
Mutant versus wild	2.57 (0.58–11.33)	0.195	5.76 (1.21–27.48)	0.013*
*EXT2*				
Mutant versus wild	3.88 (0.87–17.28)	0.055	4.74 (1.00–22.44)	0.030*
*PRDM1*				
Mutant versus wild	4.62 (1.04–20.52)	0.027*	2.04 (0.26–16.15)	0.491
TGF‐*β*				
Mutant versus wild	2.47 (0.93–6.60)	0.062	3.60 (1.01–12.78)	0.034*

Abbreviations: chemo, chemotherapy; CI, confidence interval; combination, chemotherapy plus bevacizumab or cetuximab; HR, hazard ratio; M0, without metastasis; M1, with metastasis; OS, overall survival; PFS, progression‐free survival.

** < 0.01. * < 0.05.

**FIGURE 3:**
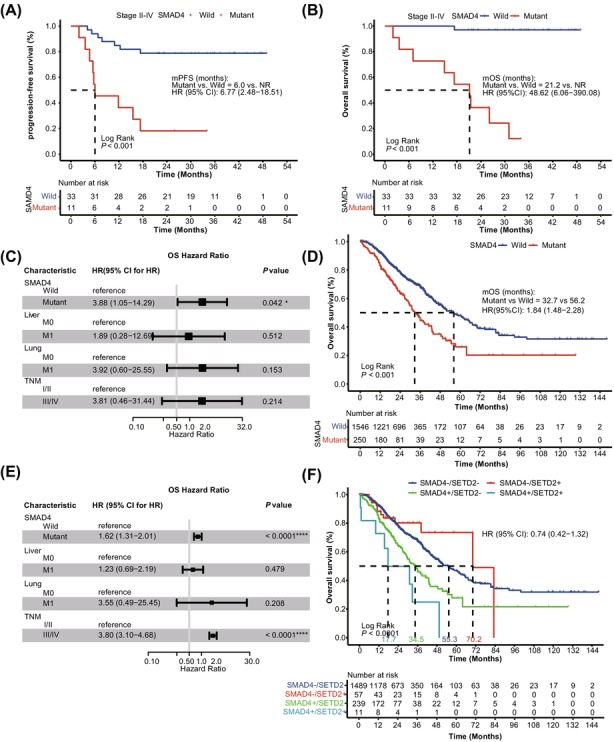
Prognostic implications of *SMAD4* mutations in CRC patients. (A, B) Kaplan–Meier curve of progression‐free survival (A) and overall survival (B) in patients with or without *SMAD4* alterations, excluding stage I CRC cases in the internal cohort. (C) Multivariate Cox analysis of overall survival in the internal cohort, including *SMAD4* mutation status, liver metastasis, lung metastasis, and TNM stage. (D) Kaplan–Meier curve of overall survival in patients with or without *SMAD4* alterations in the external cohort. (E) Multivariate Cox analysis of overall survival in the external cohort, including *SMAD4* mutation status, liver metastasis, lung metastasis, and TNM stage. (F) Kaplan–Meier curve of overall survival in the external cohort comparing four groups based on *SMAD4* and *SETD2* mutation status. A log‐rank test was used to compare overall survival or progression‐free survival between groups. *p*‐values < 0.05 were considered statistically significant. *p < 0.05, ****p < 0.0001. CI, confidence interval; HR, hazard ratio; M0, without metastasis; M1, with metastasis; mOS, median overall survival; mPFS, median progression‐free survival; NR, not reached.

Among genetic alterations, we focused on components of the TGF‐*β* signaling pathway, which plays a pivotal role in CRC tumorigenesis and progression. Notably, *SMAD4*, a central mediator of this pathway and one of the most frequently mutated genes in our cohort, was strongly associated with adverse outcomes. *SMAD4* mutations were associated with a trend toward shorter PFS (HR = 2.20, 95% CI 0.80–6.06, *p* = 0.118, Figure 3A) and a significantly reduced OS (HR = 3.68, 95% CI 1.06–12.73, *p* = 0.027, Table 2), indicating *SMAD4* as an important marker for diminished survival. After excluding stage I patients, the association of *SMAD4* mutations with both PFS (HR = 6.77, 95% CI 2.48–18.51, *p* < 0.001, Figure 3A) and OS (HR = 48.62, 95% CI 6.06–390.08, *p* < 0.001, Figure 3B) became even more significant.

Beyond TGF‐*β* pathway components, several other genetic factors were associated with adverse outcomes. Specifically, mutations in *AMER1* (HR = 5.76, 95% CI 1.21–27.48, *p* = 0.013) and *EXT2* (HR = 4.74, 95% CI 1.00–22.44, *p* = 0.030) were significantly associated with reduced OS, while *PRDM1* mutation was significantly correlated with shorter PFS (HR = 4.62, 95% CI 1.04–20.52, *p* = 0.027, Table 2).

### 3.6 The Independent Prognostic Role of *SMAD4* in CRC

Building on the univariate findings linking TGF‐*β* signaling pathway mutations to adverse prognosis, we next sought to determine whether *SMAD4* mutations exert an independent prognostic effect. Accordingly, we performed a multivariate analysis including *SMAD4* status, as well as clinical covariates with the *p*‐value < 0.15 in the univariate analyses, namely liver metastases, lung metastases, and the TNM stage. The results confirmed that *SMAD4* remained an independent predictor of poorer OS (HR = 3.88, 95% CI 1.05–14.29, *p* = 0.042, Figure 3C).

We further validated our findings in an external cohort of 1796 patients from the MSK and TCGA datasets accessed via cBioPortal (https://www.cbioportal.org). Clinical and pathological characteristics were comparable to our cohort (Table S2). In the univariate regression analysis, *SMAD4* mutations were identified as a significant predictor of poorer OS, with median OS (mOS) markedly shorter in mutated cases compared to wild‐type (32.7 months vs. 56.2 months, HR = 1.84, 95% CI 1.48–2.28, *p* < 0.0001, Figure 3D). Multivariate analysis confirmed *SMAD4* as an independent prognostic marker for OS (HR = 1.62, 95% CI 1.31–2.01, *p* < 0.0001, Figure 3E). Additionally, advanced TNM stage (III/IV) was independently associated with poorer OS (HR = 3.81, 95% CI 3.10–4.68, *p* < 0.001, Figure 3E). Notably, mutations in *SMAD4* within our cohort were predominantly clustered in the functional MH2 domain, including the known hotspot p.R361H/C (Figure S2A), a pattern consistent with the large‐scale MSK and TCGA datasets (Figure S2B).

### 3.7 The Combined Prognostic Values of *SMAD4* and *SETD2*

Given previous evidence suggesting that *SETD2* loss exacerbates genomic instability and accelerates tumor progression, particularly in *SMAD4*‐deficient CRC [29], we further explored the combined prognostic impact of these two genes. Patients were stratified into four groups according to their mutational profiles: dual mutants (*SMAD4*+/*SETD2*+), *SETD2*‐only mutants (*SMAD4*+/*SETD2*−), *SMAD4*‐only mutants (*SMAD4−*/*SETD2*+), and wild‐type for both genes (*SMAD4*−/*SETD2−*). Due to the small sample size and very limited number of dual‐mutant cases (*n* = 3) in the internal cohort, we focused on the external validation cohort. In this larger cohort, *SMAD4*+/*SETD2*+ patients exhibited shorter mOS across the whole cohort (17.7 months), significantly lower than the other groups (*p* < 0.0001, Figure 3F).

### 3.8 Prognostic Significance of Clinical and Genetic Biomarkers in LCC and RCC

Having established *SMAD4* as a significant prognostic factor in the overall CRC cohort, we next examined its relevance within LCC and RCC subgroups. In both subgroups, *SMAD4* mutations consistently showed a trend toward poorer OS, though not reaching statistical significance (LCC: HR = 3.67, 95% CI 0.82–16.49, *p* = 0.069; RCC: HR = 5.88, 95% CI 0.53–65.03, *p* = 0.101, Table S3).

Regarding clinical factors, univariate analysis revealed that lung metastasis was associated with poorer OS (HR = 6.87, 95% CI 1.32–35.81, *p* = 0.008) in LCC, whereas liver metastasis was associated with worse OS (HR = 7.99, 95% CI 0.72–88.13, *p* = 0.044) in RCC (Table S4), highlighting the location‐specific prognostic relevance of metastatic spread.

**FIGURE 4:**
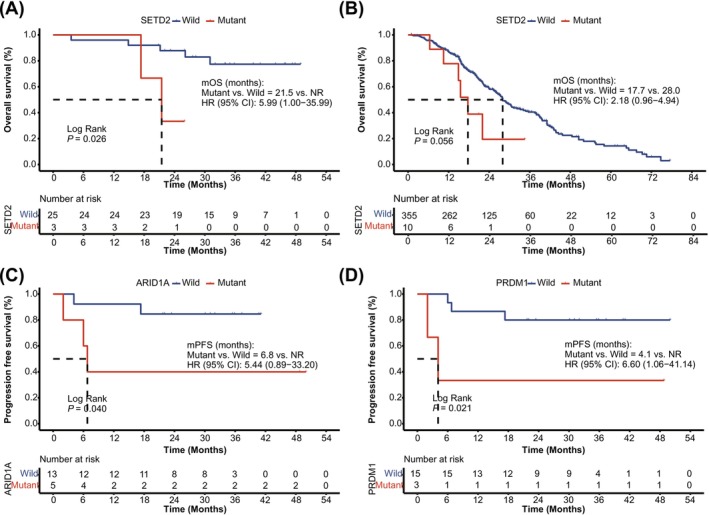
Prognostic relevance of molecular biomarkers in LCC and RCC. (A) Kaplan–Meier curve of overall survival in patients with or without *SETD2* alterations in the internal LCC cohort (A) and in the external LCC cohort (B). (C) Kaplan–Meier curve of progression‐free survival in patients with or without *ARID1A* alterations in the internal RCC cohort. (D) Kaplan–Meier curve of progression‐free survival in patients with or without *PRDM1* alterations in the internal RCC cohort. A log‐rank test was used to compare overall survival or progression‐free survival between groups. *p*‐values < 0.05 were considered statistically significant. CI, confidence interval; HR, hazard ratio; mOS, median overall survival; mPFS, median progression‐free survival; NR, not reached.

Distinct molecular prognostic markers were identified for each subgroup. In LCC, *SETD2* mutations were clearly linked to shorter OS (*p* = 0.026, Figure 4A), a finding supported by a trend toward worse OS in the external cohort (*p* = 0.056, Figure 4B). The *SETD2* mutation spectrum in LCC was characterized by a predominance of loss‐of‐function events (nonsense and frameshift) alongside a single missense mutation, suggesting functional impairment. In RCC, *ARID1A* and *PRDM1* mutations were associated with worse PFS (*p* = 0.040 and 0.021, respectively, Figure 4C,D). Notably, all identified *ARID1A* alterations were loss‐of‐function events, comprising four frameshift and one nonsense mutation.

## 4 Discussion

Our study elucidates critical insights into the differential prognostic impacts of clinical and genetic biomarkers in CRC, specifically focusing on the distinctions between LCC and RCC. This highlights the inherent heterogeneity of CRC based on tumor location. Notably, RCC exhibited a higher prevalence of *BRAF* mutations and greater enrichment of PI3K, HDR, and MMR pathway alterations. Furthermore, both *SMAD4* and TGF‐*β* signaling pathways emerged as prognostic markers associated with poorer survival, especially *SMAD4*, which was independently associated with adverse outcomes, highlighting its potential utility in personalized risk stratification and therapeutic decision‐making. Distinct molecular prognostic markers were identified, with *SETD2* mutations linked to poorer outcomes in LCC and *ARID1A* and *PRDM1* mutations impacting RCC. Importantly, our analysis indicated that concurrent *SMAD4* and *SETD2* mutations confer especially poor prognosis. These findings underscore the value of genetic mutations in informing personalized treatment strategies.

The identification of *SMAD4* as an independent prognostic marker across internal and external cohorts underscores its critical role in disease biology and patient stratification. In both LCC and RCC subgroups, *SMAD4* mutations showed a consistent trend toward poorer outcomes, although statistical significance was not reached due to limited sample size. As a tumor‐suppressor gene located on chromosome 18q21 [6, 30], *SMAD4* mutations are present in 5.0%–24.2% of CRC cases [31]. Loss of *SMAD4* disrupts TGF‐*β* signaling, and has been associated with tumor progression, metastasis, and reduced chemosensitivity [32, 33, 34, 35]. Consistent with our findings regarding poor survival, *SMAD4* alterations have been widely associated with increased tumor aggressiveness and a poly‐metastatic potential in CRC. Specifically, *SMAD4* loss or mutation appears to play a critical role in promoting a poly‐metastatic phenotype, thereby serving as a negative predictor of oligo‐metastatic behavior in both hepatic [36] and pulmonary [37] metastases. Recent evidence further underscores the biological significance of these alterations in distinguishing aggressive CRC phenotypes, noting that the absence of *SMAD4* variants is a hallmark of patients who maintain indolent, organ‐limited disease [38]. Collectively, these studies identify *SMAD4* as a crucial molecular determinant of metastatic heterogeneity. Beyond promoting metastasis, *SMAD4* deficiency confers treatment resistance. It reduces the efficacy of 5‐FU through PI3K/Akt pathway and survivin‐mediated cell cycle regulation [39]. In addition, *SMAD4* deficiency impairs the response to cetuximab [40] and alters the immune landscape by modulating cytokine production, such as IL‐10, thereby affecting T helper cells and CD8+ T cell differentiation [41]. Our findings reinforce the prognostic significance of *SMAD4* in CRC, suggesting its utility for refining risk stratification and informing targeted therapeutic strategies for high‐risk patients, including TGF‐*β* pathway inhibitors or immunotherapies.

When comparing the molecular landscapes of LCC and RCC, distinctive divergence is evident, further reinforcing and expanding upon previously established findings [15, 42, 43, 44]. RCCs not only display a higher prevalence of MSI‐H [15] but are also significantly associated with *BRAF* mutations [44, 45, 46], particularly the *BRAF* V600E, which are mutually exclusive with *APC* mutations. Moreover, RCC demonstrates heightened activities in HDR and MMR pathways compared to LCCs, suggesting potential benefits from immunotherapeutic interventions over conventional cytotoxic treatments [15, 46]. Our study suggests potential novel interactions, such as the mutual exclusivity between MSI‐H/*MED12* and *TP53*, which may have prognostic or therapeutic relevance. These findings warrant further investigation in larger and prospective cohorts to clarify their impact on personalized treatment strategies.

Distinct metastatic patterns significantly impact the prognoses of LCC and RCC, aligning with their respective molecular characteristics. Our study indicates that lung metastases in LCC significantly worsen prognosis by reducing overall survival, while liver metastases in RCC adversely affect survival outcomes. These differences may be rooted in the divergent embryological origins and vascular drainage systems of LCC and RCC, influencing immune evasion and tumor microenvironment interactions. We speculate that lung metastases might promote hypoxic adaptations in LCC, whereas liver metastases could damage the liver's immunosuppressive properties in RCC. At the molecular level, LCC and RCC exhibit site‐specific biomarkers, with *SETD2* being prognostic for LCC and *ARID1A*/*PRDM1* serving as predictive markers in RCC. *SETD2* encodes a histone methyltransferase crucial for H3K36 trimethylation [47, 48]. Loss of *SETD2* promotes genomic instability and accelerates tumor progression, particularly in the context of *SMAD4*‐deficient CRC [29]. Extending these observations, our study suggests that patients harboring concurrent *SMAD4* and *SETD2* mutations may experience a significantly poorer prognosis than those with single or no mutations. This suggests that disruption of both TGF‐*β* signaling (via *SMAD4* loss) and chromatin remodeling (via *SETD2* loss) drives more aggressive disease biology in CRC. These results underscore the clinical relevance of integrated molecular profiling, since identifying patients with concurrent *SMAD4* and *SETD2* alterations could aid in the early identification of particularly high‐risk individuals. Deleterious variations of *ARID1A* are correlated with poor prognosis [49] and both intrinsic and acquired resistance to cetuximab therapy in CRC [50]. *PRDM1* mediates stress‐induced ribosomal dysfunction, driving tumor cell survival and stemness via insulin‐like growth factor receptor pathway reprogramming [51]. The distinct metastatic patterns and functional roles of these biomarkers highlight the complex interplay between genetic alterations and the tumor microenvironment and underscore the importance of mechanistic investigation to inform more individualized surveillance and therapeutic strategies in CRC.

Despite the robust insights provided, our study has several limitations inherent to its retrospective design. First, the non‐randomized data collection introduces potential selection and information biases, as well as confounding variables, such as heterogeneous treatment regimens and varying follow‐up durations, which may affect the generalizability of the findings. Second, the inclusion of patients across diverse disease stages (I‐IV) represents an additional limitation. While stage was accounted for in multivariable analyses, stage‐specific molecular differences and varying prognostic baselines could influence the interpretation of the observed associations. Third, the relatively limited sample size precluded further subdivision of tumor sites. While emerging evidence suggests that a molecular continuum from cecum to rectum may better explain mutational and metabolic differences than the binary left–right classification [2, 52, 53, 54], the LCC versus RCC dichotomy remains the current standard for clinical decision‐making. Future prospective studies with larger cohorts and stage‐stratified analyses are essential to validate these prognostic markers and refine their clinical utility.

## 5 Conclusions

In conclusion, our study provides a comprehensive analysis of the mutational landscape of CRC and distinct molecular and prognostic landscapes of LCC and RCC, suggesting a potential need for location‐specific therapeutic strategies. The identification of *SMAD4* and TGF‐*β* as pivotal prognostic markers, alongside the differential metastatic impacts on survival, offers valuable data to improve the stratification and management of CRC patients. Our findings support a refined understanding of CRC heterogeneity, potentially informing the development of more effective, individualized treatments.

## Author Contributions

**Wenlei Zhao:** conceptualization (equal), data curation (equal), investigation (equal), methodology (equal), writing – review and editing (equal). **Yuxuan Qiu:** conceptualization (equal), data curation (equal), investigation (equal), methodology (equal), writing – review and editing (equal). **Na Bai:** data curation (equal), formal analysis (lead), investigation (equal), writing – review and editing (equal). **Chunhong He:** validation (equal), writing – original draft (equal), writing – review and editing (equal). **Jiani C. Yin:** validation (equal), writing – original draft (equal), writing – review and editing (equal). **Qianru Xu:** resources (equal), writing – review and editing (equal). **Kaiyu Jian:** resources (equal), writing – review and editing (equal). **Baolei Jia:** resources (equal), writing – review and editing (equal). **Lin Jiang:** resources (equal), writing – review and editing (equal). **Feng Liang:** conceptualization (equal), methodology (equal), project administration (lead), supervision (equal), writing – review and editing (equal).

## Ethics Statement

This study was approved by the Research Ethics Board of the Fifth Medical Center of the Chinese People's Liberation Army General Hospital (No. ky‐2021‐1‐2) and was conducted in accordance with the Declaration of Helsinki. Written informed consent was obtained from all the participants.

## Conflicts of Interest

Na Bai, Chunhong He, and Jiani C. Yin are employees of Nanjing Geneseeq Technology Inc. All other authors declare that they have no conflicts of interest regarding the research presented in this manuscript.

## Supporting information


**Figure S1:** Mutational landscape and pathway alterations in LCC and RCC.

**Figure S2:** Lollipop plots of *SMAD4* mutations.

**Table S1:** The list of 425 genes included in the GeneseeqPrime panel.

**Table S2:** Clinicopathologic and molecular characteristics of the external study cohort.

**Table S3:** The prognostic relevance of *SMAD4* in the subgroups.

**Table S4:** Prognostic relevance of clinical characteristics in LCC and RCC.

## Data Availability

The data that support the findings of this study are available from the corresponding author upon reasonable request. The external cohort for validation is openly available and accessible in cBioPortal (https://www.cbioportal.org).
