# Supplementary material for: Distinct Molecular and Prognostic Profiles of Left‐ and Right‐Sided Colorectal Cancer Revealed by NGS Analysis: The Role of SMAD4 and SETD2 Mutations
Source: Cancer Med. 2026 Jan 21;15(1):e71534. doi: 10.1002/cam4.71534 (PMC12820718; doi:10.1002/cam4.71534)
Supplement: Supplementary file 1 — Figure S1: Mutational landscape and pathway alterations in LCC and RCC. [file CAM4-15-e71534-s005.docx]

**Figure S1**


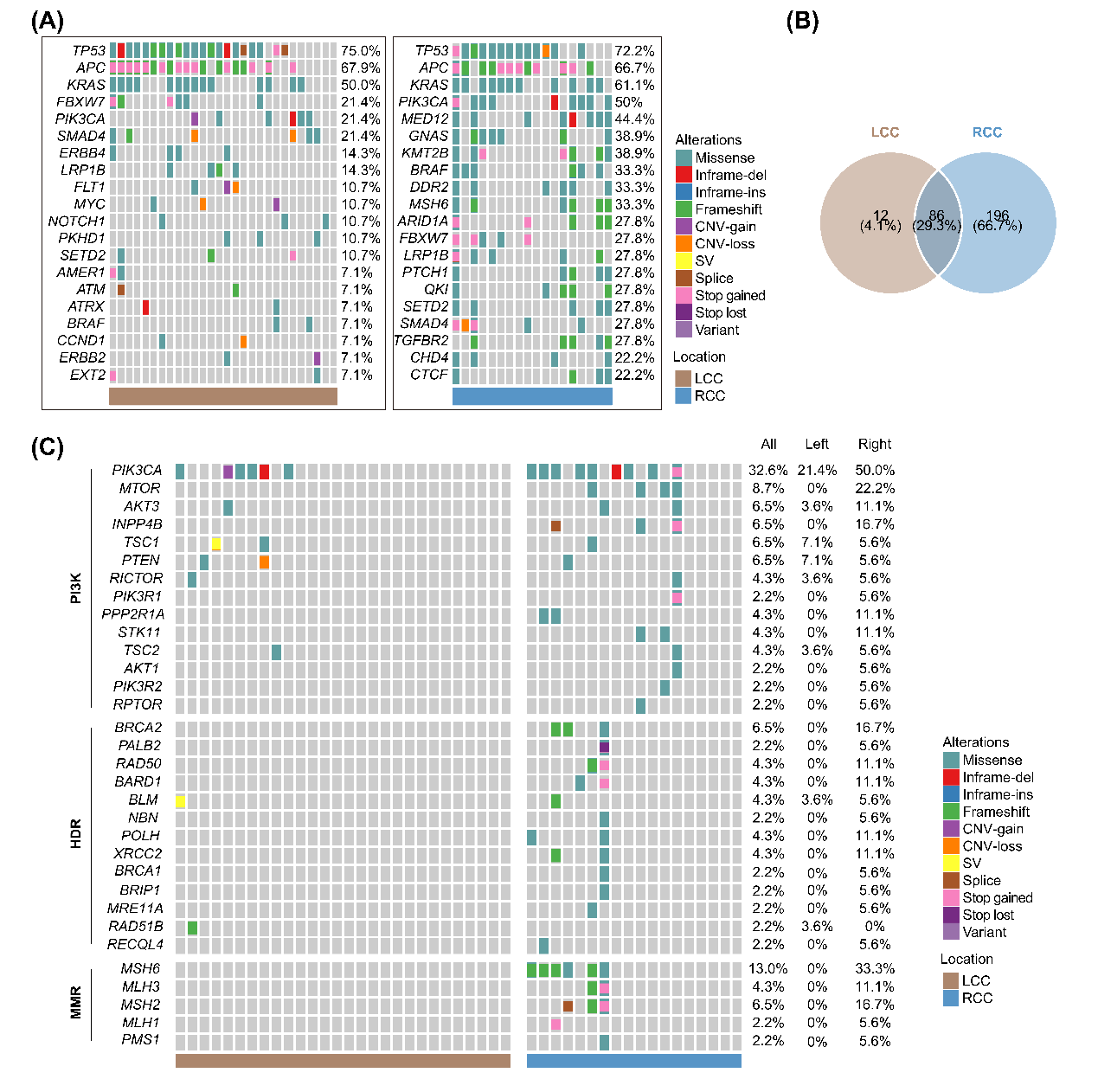


**Clinicopathologic and molecular characteristics of the external study cohort.**

**(A)** The detailed mutational landscape for LCC and RCC patients. LCC is covered in the left panel, RCC in the right. The right-side color legend distinguishes between different mutation types. Each column represents an individual patient in the cohort.

**(B)** Venn diagram summary of mutated genes derived from both LCC and RCC groups.

**(C)** Analysis of genetic alterations in the PI3K, HDR, and MMR pathways in LCC and RCC groups. Mutation frequencies are annotated next to the respective panels, with mutation types indicated by the color legend on the right. Each column represents an individual patient in the cohort.

CNV, copy number variation; SV, structural variation; LCC, left-sided colorectal cancer; RCC, right-sided colorectal cancer; PI3K, phosphatidylinositol-3-kinase; HDR, homologous-dependent recombination; MMR, mismatch repair.
