# Supplementary material for: Distinct Molecular and Prognostic Profiles of Left‐ and Right‐Sided Colorectal Cancer Revealed by NGS Analysis: The Role of SMAD4 and SETD2 Mutations
Source: Cancer Med. 2026 Jan 21;15(1):e71534. doi: 10.1002/cam4.71534 (PMC12820718; doi:10.1002/cam4.71534)
Supplement: Supplementary file 2 — Figure S2: Lollipop plots of SMAD4 mutations. [file CAM4-15-e71534-s003.docx]

**Figure S2**


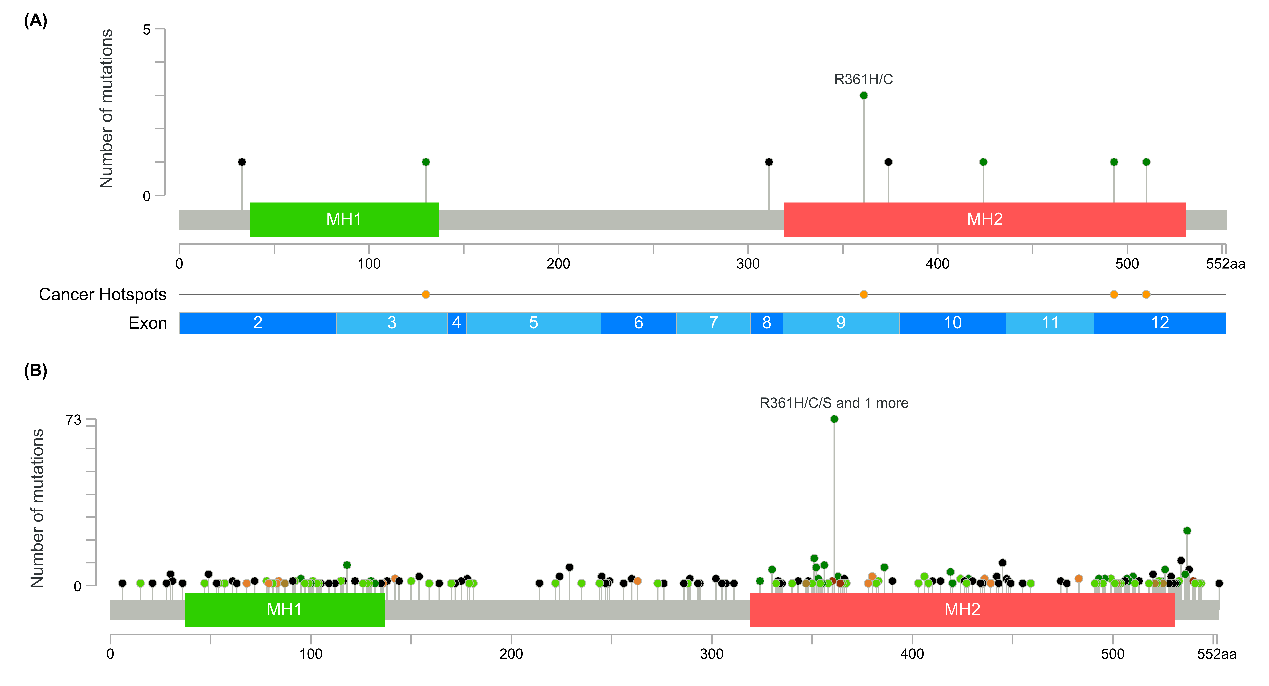


**Lollipop plots of *SMAD4* mutations.**

**(A-B)** Distribution of SMAD4 variants in the internal cohort **(A)** and in the external TCGA cohort **(B)**. The height of the stick represents the number of samples harboring the specific mutation.
