# Supplementary material for: Distinct Molecular and Prognostic Profiles of Left‐ and Right‐Sided Colorectal Cancer Revealed by NGS Analysis: The Role of SMAD4 and SETD2 Mutations
Source: Cancer Med. 2026 Jan 21;15(1):e71534. doi: 10.1002/cam4.71534 (PMC12820718; doi:10.1002/cam4.71534)
Supplement: Supplementary file 3 — Table S1: The list of 425 genes included in the GeneseeqPrime panel. [file CAM4-15-e71534-s002.docx]

**Table S1**

**The list of 425 genes included in the GeneseeqPrime® panel.**

| *ABCB1* | *ABCC2* | *ADH1B* | *AIP* | *AKT1* | *AKT2* | *AKT3* | *ALDH2* | *ALK* | *AMER1* | *APC* | *AR* | *ARAF* | *ARID1A* | *ARID1B* | *ARID2* | *ARID5B* |
| --- | --- | --- | --- | --- | --- | --- | --- | --- | --- | --- | --- | --- | --- | --- | --- | --- |
| *ASCL4* | *ASXL1* | *ATF1* | *ATIC* | *ATM* | *ATR* | *ATRX* | *AURKA* | *AURKB* | *AXIN2* | *AXL* | *B2M* | *BAD* | *ADGRB3* | *BAK1* | *BAP1* | *BARD1* |
| *BAX* | *BCL2* | *BCL2L11* | *BCR* | *BIRC3* | *BLM* | *BMPR1A* | *BRAF* | *BRCA1* | *BRCA2* | *BRD4* | *BRIP1* | *BTG2* | *BTK* | *BUB1B* | *EMSY* | *CASP8* |
| *CBL* | *CBLB* | *CCND1* | *CCNE1* | *CD274* | *CD74* | *CDA* | *CDC73* | *CDH1* | *CDK10* | *CDK12* | *CDK4* | *CDK6* | *CDK8* | *CDKN1A* | *CDKN1B* | *CDKN1C* |
| *CDKN2A* | *CDKN2B* | *CDKN2C* | *CEBPA* | *CEP57* | *CHD4* | *CHD8* | *CHEK1* | *CHEK2* | *CREBBP* | *CRKL* | *CSF1R* | *CTCF* | *CTLA4* | *CTNNB1* | *CUL3* | *CUX1* |
| *CXCR4* | *CYLD* | *CYP19A1* | *CYP2A13* | *CYP2A6* | *CYP2A7* | *CYP2B6* | *CYP2C19* | *CYP2C9* | *CYP2D6* | *CYP3A4* | *CYP3A5* | *CYSLTR2* | *DAXX* | *DDR2* | *DENND1A* | *DHFR* |
| *DICER1* | *DLL3* | *DNMT3A* | *DOT1L* | *DPYD* | *DTL* | *DUSP2* | *EGFR* | *EIF1AX* | *EP300* | *EPAS1* | *EPCAM* | *EPHA2* | *EPHA3* | *EPHA5* | *ERBB2* | *ERBIN* |
| *ERBB3* | *ERBB4* | *ERCC1* | *ERCC2* | *ERCC3* | *ERCC4* | *ERCC5* | *ESR1* | *ETV1* | *ETV4* | *ETV5* | *ETV6* | *EWSR1* | *EXT1* | *EXT2* | *EZH2* | *EZR* |
| *FANCA* | *FANCC* | *FANCD2* | *FANCE* | *FANCF* | *FANCG* | *FANCI* | *FANCL* | *FANCM* | *FAT1* | *FBXW7* | *FGF19* | *FGFR1* | *FGFR2* | *FGFR3* | *FGFR4* | *FH* |
| *FLCN* | *FLT1* | *FLT3* | *FLT4* | *FOXA1* | *FOXL2* | *FOXP1* | *FRG1* | *GATA1* | *GATA2* | *GATA3* | *GATA4* | *GATA6* | *GNA11* | *GNAQ* | *GNAS* | *GRIN2A* |
| *GRM3* | *GRM8* | *GSTM1* | *GSTM4* | *GSTP1* | *GSTT1* | *HDAC2* | *HDAC9* | *HGF* | *HLA-A* | *HNF1A* | *HNF1B* | *HRAS* | *IDH1* | *IDH2* | *IFNA6* | *IFNB1* |
| *IFNE* | *IFNG* | *IFNGR1* | *IFNGR2* | *IGF1R* | *IGF2* | *IKBKE* | *IKZF1* | *IL7R* | *INPP4B* | *IRF2* | *JAK1* | *JAK2* | *JAK3* | *JARID2* | *JUN* | *KDM5A* |
| *KDR* | *KEAP1* | *KIF1B* | *KIT* | *KITLG* | *KLLN* | *KMT2A* | *KMT2B* | *KMT2C* | *KMT2D* | *KRAS* | *LHCGR* | *LMO1* | *LRP1B* | *LYN* | *LZTR1* | *MAP2K1* |
| *MAP2K2* | *MAP2K4* | *MAP3K1* | *MAP3K4* | *MAX* | *MCL1* | *MDM2* | *MDM4* | *MECOM* | *MED12* | *MEF2B* | *MEN1* | *MET* | *MGMT* | *MITF* | *MLH1* | *MLH3* |
| *MLLT1* | *MLLT3* | *AFDN* | *MPL* | *MRE11* | *MSH2* | *MSH6* | *MTHFR* | *MTOR* | *MUTYH* | *MYC* | *MYCL* | *MYCN* | *MYD88* | *MYH9* | *NAT1* | *NBN* |
| *NCOR1* | *NF1* | *NF2* | *NFE2L2* | *NFKBIA* | *NKX2-1* | *NOTCH1* | *NOTCH2* | *NOTCH3* | *NPM1* | *NQO1* | *NRAS* | *NRG1* | *NSD1* | *NTRK1* | *NTRK2* | *NTRK3* |
| *NUTM1* | *PAK3* | *PALB2* | *PALLD* | *PRKN* | *PARP1* | *PARP2* | *PAX5* | *PBRM1* | *PDCD1* | *PDCD1LG2* | *PDE11A* | *PDGFRA* | *PDGFRB* | *PDK1* | *PGR* | *PHOX2B* |
| *PIK3C3* | *PIK3CA* | *PIK3CD* | *PIK3R1* | *PIK3R2* | *PKHD1* | *PLAG1* | *PLCB4* | *PLK1* | *PMS1* | *PMS2* | *POLD1* | *POLD3* | *POLE* | *POLH* | *POT1* | *PPARD* |
| *PPP2R1A* | *PRDM1* | *PREX2* | *PRF1* | *PRKACA* | *PRKAR1A* | *PRKCI* | *PRKDC* | *PRSS1* | *PRSS3* | *PTCH1* | *PTEN* | *PTK2* | *PTPN11* | *PTPN13* | *QKI* | *RAC1* |
| *RAC3* | *RAD50* | *RAD51* | *RAD51B* | *RAD51C* | *RAD51D* | *RAD54L* | *RAF1* | *RARA* | *RARG* | *RASGEF1A* | *RB1* | *RECQL4* | *RELN* | *RET* | *RHOA* | *RICTOR* |
| *RNF43* | *ROS1* | *RPTOR* | *RRM1* | *RUNX1* | *RUNX1T1* | *SBDS* | *SDC4* | *SDHA* | *SDHB* | *SDHC* | *SDHD* | *SEPTIN9* | *SETBP1* | *SETD2* | *SF3B1* | *SGK1* |
| *SKP2* | *SLC34A2* | *SLC3A2* | *SMAD2* | *SMAD3* | *SMAD4* | *SMAD7* | *SMARCA4* | *SMARCB1* | *SMO* | *SOCS1* | *SOS1* | *SOX2* | *SPOP* | *SPRED1* | *SPRY4* | *SRC* |
| *SRSF2* | *SRY* | *STAG2* | *STAT3* | *STK11* | *STMN1* | *SUFU* | *TACC3* | *TAP1* | *TAP2* | *TEK* | *TEKT4* | *TERC* | *TERT* | *TET2* | *TGFBR2* | *THADA* |
| *TMEM127* | *TMPRSS2* | *TNFAIP3* | *TNFRSF11A* | *TNFRSF14* | *TNFRSF19* | *TNFSF11* | *TOP1* | *TOP2A* | *TP53* | *TP63* | *TPMT* | *TSC1* | *TSC2* | *TSHR* | *TTF1* | *TUBB3* |
| *TYMS* | *U2AF1* | *UGT1A1* | *VAMP2* | *VEGFA* | *VHL* | *WAS* | *CCN6* | *WRN* | *WT1* | *XPA* | *XPC* | *XRCC1* | *XRCC2* | *YAP1* | *ZNF217* | *ZNF703* |
