# Supplementary material for: Distinct Molecular and Prognostic Profiles of Left‐ and Right‐Sided Colorectal Cancer Revealed by NGS Analysis: The Role of SMAD4 and SETD2 Mutations
Source: Cancer Med. 2026 Jan 21;15(1):e71534. doi: 10.1002/cam4.71534 (PMC12820718; doi:10.1002/cam4.71534)
Supplement: Supplementary file 4 — Table S2: Clinicopathologic and molecular characteristics of the external study cohort. [file CAM4-15-e71534-s001.docx]

**Table S2**

**Clinicopathologic and molecular characteristics of the external study cohort.**

| **Characteristic** | **All (*n* = 1,796)** |
| --- | --- |
| **Age, *n* (%)** |  |
| ≥ 60 | 817 (45.5) |
| < 60 | 400 (22.3) |
| **Sex, *n* (%)** |  |
| Male | 953 (53.1) |
| Female | 843 (46.9) |
| **Differentiation, *n* (%)** |  |
| Poorly differentiated | 120 (6.7) |
| Poorly-moderately differentiated | 5 (0.5) |
| Moderately differentiated | 328 (18.3) |
| Well differentiated | 6 (0.5) |
| **Pathological stage, *n* (%)** |  |
| I | 221 (12.3) |
| II | 446 (24.8) |
| III | 445 (24.8) |
| IV | 482 (26.8) |
| **ECOG score, *n* (%)** |  |
| 0-1 | 356 (19.8) |
| 2/other | 27 (1.5) |
| **MS status, *n* (%)** |  |
| MSI-H | 107 (6.0) |
| MSS | 938 (52.2) |
| **TMB, *n* (%)** |  |
| TMB-H | 220 (12.2) |
| TMB-L | 1093 (60.9) |
| **Primary tumor location, *n* (%)** |  |
| LCC | 365 (20.3) |
| RCC | 199 (11.1) |

ECOG, Eastern Cooperative Oncology Group; Chemo, chemotherapy; MS, microsatellite; MSI-H, high microsatellite instability; MSS, microsatellite stable; TMB, tumor mutation burden; TMB-H, high tumor mutation burden; TMB-L, low tumor mutation burden; LCC, left-sided colorectal cancer; RCC, right-sided colorectal cancer.
