# Supplementary material for: Distinct Molecular and Prognostic Profiles of Left‐ and Right‐Sided Colorectal Cancer Revealed by NGS Analysis: The Role of SMAD4 and SETD2 Mutations
Source: Cancer Med. 2026 Jan 21;15(1):e71534. doi: 10.1002/cam4.71534 (PMC12820718; doi:10.1002/cam4.71534)
Supplement: Supplementary file 5 — Table S3: The prognostic relevance of SMAD4 in the subgroups. [file CAM4-15-e71534-s004.docx]

**Table S3**

**The prognostic relevance of *SMAD4* in the subgroups.**

| **Location** | ***n* (%)** | **OS** | |
| --- | --- | --- | --- |
|  |  | **HR (95% CI)** | ***P* value** |
| **LCC** | 6 (21.4) | 3.67 (0.82-16.49) | 0.069 |
| **RCC** | 5 (27.8) | 5.88 (0.53-65.03) | 0.101 |

LCC, left-sided colorectal cancer; RCC, right-sided colorectal cancer; OS, overall survival; HR, hazard ratio; CI, confidence interval.
