# Supplementary material for: Distinct Molecular and Prognostic Profiles of Left‐ and Right‐Sided Colorectal Cancer Revealed by NGS Analysis: The Role of SMAD4 and SETD2 Mutations
Source: Cancer Med. 2026 Jan 21;15(1):e71534. doi: 10.1002/cam4.71534 (PMC12820718; doi:10.1002/cam4.71534)
Supplement: Supplementary file 6 — Table S4: Prognostic relevance of clinical characteristics in LCC and RCC. [file CAM4-15-e71534-s006.docx]

**Table S4**

**Prognostic relevance of clinical characteristics in LCC and RCC.**

| **Characteristics** | **LCC PFS** | | **LCC OS** | | **RCC PFS** | | **RCC OS** | | |
| --- | --- | --- | --- | --- | --- | --- | --- | --- | --- |
|  | **HR**  **(95% CI)** | ***P***  **value** | **HR**  **(95% CI)** | ***P***  **value** | **HR**  **(95% CI)** | ***P* value** | **HR**  **(95% CI)** | ***P* value** |  |
| **Sex** |  |  |  |  |  |  |  |  |  |
| Male vs. Female | 0.59  (0.15-2.30) | 0.445 | 0.63  (0.12-3.27) | 0.582 | 2.15  (0.36-12.87) | 0.392 | 2.64  (0.24-29.14) | 0.410 |  |
| **Age** |  |  |  |  |  |  |  |  |  |
| ≥ 60 vs. <60 | 0.49  (0.14-1.73) | 0.255 | 0.32  (0.06-1.65) | 0.151 | 0.64  (0.11-3.86) | 0.628 | 0.84  (0.08-9.23) | 0.884 |  |
| **TNM** |  |  |  |  |  |  |  |  |  |
| III+IV vs. I+II | 2.23  (0.47-10.51) | 0.300 | 3.03  (0.37-25.18) | 0.280 | >1000  (0-Inf) | 0.081 | >1000  (0-Inf) | 0.200 |  |
| **Liver metastasis** |  |  |  |  |  |  |  |  |  |
| M1 vs. M0 | 1.26  (0.16-9.96) | 0.827 | 1.88  (0.23-15.70) | 0.552 | 3.72  (0.42-33.36) | 0.207 | 7.99  (0.72-88.13) | 0.044* |  |
| **Lung metastasis** |  |  |  |  |  |  |  |  |  |
| M1 vs. M0 | 4.25  (0.88-20.68) | 0.051* | 6.87  (1.32-35.81) | 0.008* | 1.74  (0.19-15.56) | 0.618 | 3.74  (0.34-41.31) | 0.247 |  |
| **Lymph node** |  |  |  |  |  |  |  |  |  |
| N1 vs. N0 | 1.83  (0.39-8.64) | 0.438 | 2.45  (0.30-20.38) | 0.391 | >1000  (0-Inf) | 0.081 | >1000  (0-Inf) | 0.200 |  |
| **Differentiation** |  |  |  |  |  |  |  |  |  |
| Moderate/moderate-low vs. Low | 1.48  (0.19-11.88) | 0.708 | 1.04  (0.12-8.87) | 0.975 | >1000  (0-Inf) | 0.562 | >1000  (0-Inf) | 0.665 |  |

LCC, left-sided colorectal cancer; RCC, right-sided colorectal cancer; PFS, progression-free survival; OS, overall survival; HR, hazard ratio; CI, confidence interval; M1, with metastasis; M0, without metastasis; N1, with metastasis; N0, without metastasis; Inf, infinite.

* < 0.05.
